# Performance Studies on Distributed Virtual Screening

**DOI:** 10.1155/2014/624024

**Published:** 2014-06-17

**Authors:** Jens Krüger, Richard Grunzke, Sonja Herres-Pawlis, Alexander Hoffmann, Luis de la Garza, Oliver Kohlbacher, Wolfgang E. Nagel, Sandra Gesing

**Affiliations:** ^1^Center for Bioinformatics, Quantitative Biology Center, and Department of Computer Science, University of Tübingen, Sand 14, 72076 Tübingen, Germany; ^2^Technische Universität Dresden, Zellescher Weg 12-14, 01069 Dresden, Germany; ^3^Ludwig-Maximilians-Universität München, Butenandtstr aße 5-13, 81377 München, Germany; ^4^Center for Research Computing, University of Notre Dame, P.O. Box 539, Notre Dame, IN 46556, USA

## Abstract

Virtual high-throughput screening (vHTS) is an invaluable method in modern drug discovery. It permits screening large datasets or databases of chemical structures for those structures binding possibly to a drug target. Virtual screening is typically performed by docking code, which often runs sequentially. Processing of huge vHTS datasets can be parallelized by chunking the data because individual docking runs are independent of each other. The goal of this work is to find an optimal splitting maximizing the speedup while considering overhead and available cores on Distributed Computing Infrastructures (DCIs). We have conducted thorough performance studies accounting not only for the runtime of the docking itself, but also for structure preparation. Performance studies were conducted via the workflow-enabled science gateway MoSGrid (Molecular Simulation Grid). As input we used benchmark datasets for protein kinases. Our performance studies show that docking workflows can be made to scale almost linearly up to 500 concurrent processes distributed even over large DCIs, thus accelerating vHTS campaigns significantly.

## 1. Introduction 

Drug discovery is a time-consuming, risky, and expensive process. In general, it takes about 14 years until a new drug reaches the market [1]. To shorten the research cycle and to lower the failure rate, Computer-Aided Drug Design is applied in the early drug discovery phases. Virtual high-throughput screening (vHTS) is a method in Computer-Aided Drug Design that searches libraries of chemical compounds to identify active compounds. Ideally, the search and analysis are performed efficiently and result in a set of potential ligands for further experimental validation. Nowadays, an enormous number of chemical compounds are available in various databases to aid drug discovery via virtual screening such as ZINC [2]. Virtual screening supports parallelization among compounds by splitting data into distinct screening jobs. For optimal performance on DCIs, several aspects have to be considered. These can be divided into general applicable aspects and aspects dependent on the virtual screening method. The general ones reflect the users' environment for virtual screening and include the time for splitting the datasets, the actual transfer of the chunks of data to a computing node, and the overhead to manage parallel jobs. The runtime of the virtual screening method depends on the simulation method used on the assigned computing node. Thus, aspects like multicore support and the complexity of the implemented algorithm determine the performance.

Docking tools belong to the category of structure-based virtual screening methods and our approach uses the docking application IMGDock, a tool of the software framework CADDSuite (Computer-Aided Drug Design) [3]. IMGDock is a sequential tool, which evaluates ligand-receptor interactions. In this study we consider not only the docking itself, but also the whole workflow required for a vHTS campaign, which includes the preparation of receptors and ligands for the docking process (i.e., defining a binding pocket) and the parallelization of docking simulations. Docking aims at the correct placement of a ligand into the binding pocket of a receptor. The binding energy of the resulting complex is then estimated, considering the interactions between ligand and binding site. Over the past decades, several docking applications have been released and published. They can be categorized by their placement algorithm and their scoring functions. AutoDock [4], for example, relies on precomputed interaction grids and a genetic algorithm [4, 5]. In contrast FlexX first decomposes the ligand, then places the major fragment on interaction points inside the binding pocket, and finally uses a multigreedy heuristic to reconstruct the ligand [6]. The docking application IMGDock also relies on precalculated interaction grids and uses a multigreedy approach for ligand placement [3]. Scoring functions can generally be divided into three classes, although there is no strict separation and hybrid functions exist. Examples for a force field-based scoring would be AutoDock and CADDSuite. FlexX uses an empirical function, while, for example, DSX uses a knowledge-based approach [7].

The performance studies have been conducted via the workflow-enabled science gateway MoSGrid (Molecular Simulation Grid) [8–10]. It offers intuitive user interfaces for docking tools amongst other applications for molecular simulations. The science gateway supports users in managing the whole process of docking while hiding the complex underlying DCI from the user. It not only addresses usability aspects but also increases the complexity of the infrastructure to be considered in the performance studies. MoSGrid currently offers access to AutoDock Vina [4], FlexX, and CADDSuite.

Science gateways are widely used in the life sciences and chemistry community. A similar approach to MoSGrid is followed by the AutoDock portal [11], which supports docking workflows with AutoDock, whereas MoSGrid as aforementioned allows for the use of AutoDock, FlexX, and CADDSuite. Both solutions were developed on top of WS-PGRADE [11], a workflow-enabled science gateway designed for the flexible support of diverse applications and DCIs. The generic-purpose science gateway frameworks EnginFrame [12] and OGCE [13] follow a similar approach like WS-PGRADE. EnginFrame offers docking workflows via the GENIUS portal [12]. A drawback for consortiums with academic and industrial partners is its underlying business model. The docking portal BioDrugScreen [14] is developed on top of OGCE and is less flexible in its workflow support compared to the MoSGrid science gateway. Galaxy [15] is a further mature workflow-enabled science gateway but supports fewer DCIs than WS-PGRADE. Furthermore, libraries or APIs, for example, the Vine Toolkit [16] and HubZero [17], allow an easy development of DCI-enabled science gateways including custom user interfaces, but data and workflow management is often not supported. The WeNMR portal [18] can be used for molecular simulations for NMR-based structure elucidation. It provides preconfigured workflows, interfaces, and applications to handle these use cases but is not as flexible as the MoSGrid portal for creating workflows. Besides the aforementioned web-based solutions, workbenches like Taverna [19] and the Unicore Rich Client [20] are mature solutions for docking workflows. However, workbenches require installation of software on the users' computers.

Our performance studies examine the overall time from invoking a docking process via the science gateway up to receiving the results back in the science gateway in addition to measuring performance like runtimes on the computing nodes. This time frame is especially interesting since it is the time recognized by the users. Furthermore, we did not use a dedicated local compute resource but resources generally available via the science gateway to get a realistic assessment of the time users will have to wait for their results in a typical real-life setting. To our best knowledge, the investigation of the performance of the whole science gateway infrastructure and for docking workflows with split data has not been performed for other science gateways or docking methods yet.

## 2. Materials and Methods

### 2.1. Selected Structures and Docking Workflow

To study the computational performance, we used the benchmark dataset for the tyrosine-protein kinase ABL1 (PDB code 2HZI), containing 295 known active ligands and 10,885 inactive ones. DUD-E [21, 22] poses a challenge for each docking tool currently available. The dataset is of sufficient size to generate generalized benchmark data in terms of portal-based high-performance computing. In order to assess the influence of the target system itself, five smaller test sets from the DEKOIS [23, 24] test set were used (acetylcholine esterase, 1DX6; androgen receptor, 1E3G; estrogen receptor beta, 1I0G; HIV1 protease, 1HXW; and thrombin, 3RLW). Each set contains 40 active ligands and 1200 decoys.

The basic algorithm of docking tools typically includes three major steps.

1. First, many plausible structures of a complex are generated. Therefore, a huge search space has to be globally or locally investigated. Global methods scan the whole target's surface, whereas local docking can be applied in case the binding site is already identified. Both approaches have to inspect the ligand's plausible poses around a binding site, but local docking reduces the search space on the target's surface and, thus, the required computing time.
2. After generating the plausible structures, the algorithm filters out geometrically or energetically unfavorable structures.
3. Finally, the algorithm predicts the binding free energy of the remaining structures via scoring/energy functions. The assumption is that better scores imply a closer approximation of the true structure of the protein-ligand complex.

**Figure 1:**
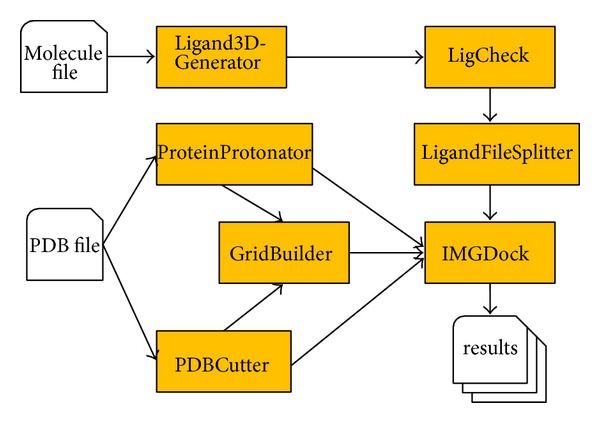
A docking workflow for CADDSuite including the preparation of the receptor and the preparation of the ligands.

Before a docking tool is invoked, it is crucial to examine whether a ligand and a target are provided in the suitable format for the applied docking software. Thus, a docking process does not only consist of the docking method itself but implies a whole workflow.  Figure 1 illustrates this workflow for CADDSuite with its docking application IMGDock.

PDBCutter is used to split a structure into its receptor and ligand parts. The extracted ligand forms the reference ligand for defining the binding pocket of the receptor. Hydrogen atoms are essential for the docking process and if hydrogen atoms are missing after the splitting task, they are added to the receptor structure via ProteinProtonator. The output of PDBCutter as well as ProteinProtonator is used in GridBuilder to build an interaction grid for the binding pocket of the receptor. The resulting grid and the receptor file set the stage to dock a set of ligands with IMGDock. The ligands have to be prepared for docking by generating 3D conformations and adding hydrogens to them (Ligand3DGenerator). Furthermore, a sanity check (LigCheck) is performed. The sanity check examines, for example, whether the ligands are properly protonated, possess suitable bond lengths, and have properly assigned binding orders. The LigandFileSplitter allows splitting the ligand file into a number of subsets and the workflow can be parallelized based on the number of splits. Finally, IMGDock is executed, which applies an empirical scoring function and consequently generates a list of ligand poses with their corresponding estimates for binding free energies.

The workflow is preconfigured in the MoSGrid science gateway and can be applied by the users to arbitrary input datasets.

### 2.2. The MoSGrid Science Gateway

Distributed science gateway infrastructures are essential in today's and tomorrow's research landscape. From the users' point of view, they provide a seamless environment where simulations are performed and data analyzed in an efficient and user-friendly way via graphical user interfaces. The underlying infrastructure is of high complexity and of growing maturity. It includes several layers: the user interface, the workflow management, the data and metadata management, and the underlying storage and compute resources. The MoSGrid science gateway is a virtual research environment for molecular chemists. It was built in the BMBF project MoSGrid and is currently further developed and operated in the SCI-BUS [25] and ER-flow [26] EU funded projects. An XSEDE project [27] is currently starting to port MoSGrid to the XSEDE infrastructure to enable US-based computational chemists to easily perform their calculations on XSEDE resources.

A key aspect of the MoSGrid design is its usability. The science gateway is based on the gUSE/WS-PGRADE portal technology, which provides services for the whole life cycle of workflows on DCIs. The Molecular Simulation Markup Language (MSML) [28] has been developed in MoSGrid and forms a metadata format for domain, job, and workflow data especially for the three main domains quantum chemistry, molecular dynamics, and docking. MSML is applied to automatically generate specifically tailored user interfaces. The user is enabled to submit simulations via intuitive user interfaces hiding the complexity of the workflow management and offering data as input using the integrated metadata management [29].

Another major aspect is that in principle storage or compute resources of any size and number can be accessed via the MoSGrid science gateway. Distributed data management is currently offered via the object-oriented file system XtreemFS [30], while the design allows the integration of various file systems like dCache [31] or iRODS [32]. These extensions are being planned to further increase the sustainability. Compute resources are made available and are handled via the scalable and mature computing middleware UNICORE [33], which is used by major infrastructures and projects like PRACE [34], XSEDE [27], EGI [35], and soon the Human Brain Project [36].

By using advanced authentication methods like Security Assertion Markup Language (SAML) [37] trust delegation assertions, MoSGrid is prepared in an optimal way for the spread of federated identity management systems like Shibboleth [38]. They enable the user to utilize the login of their home institution for other services, like the MoSGrid science gateway.

The seamless integration of computing, workflow, data, and metadata management with an easy-to-use user interface optimally supports the users in solving their highly complex and data-intensive research questions. In addition to workflows, the concept of metaworkflows has been proven to be beneficial for the users.

### 2.3. Workflows in Computational Chemistry

Workflow implementation has a long tradition in MoSGrid using the embedded graphical workflow editor of WS-PGRADE and the available DCIs. In collaboration with the SHIWA project [39], metaworkflows have been developed in MoSGrid. In the course of the collaboration, the idea came up that lots of workflows consist of subunits, which are repeated in other workflows [40]. These subunits themselves represent small workflows. In particular, for the investigation of quantum chemical questions, the workflow idea is helpful to perform the screening of large numbers of molecules.

**Figure 2:**
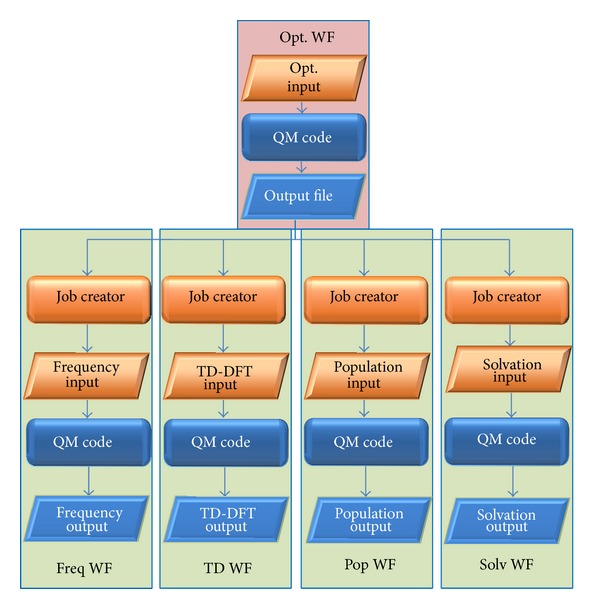
Example for a metaworkflow containing basic workflows.

The first step is always to build up the input file for a geometry optimization, which is followed then by subsequent steps of frequency calculation (freq WF), population analysis (pop WF), time-dependent DFT (TD-DFT, TD WF), and added solvation models (solv WF). For each of these subsequent jobs, the output coordinates of the optimization have to be used and inserted into a new input file. Hence, every job step represents a small basic workflow with a job definition step before job submission. The combination of these small basic workflows yields a metaworkflow (see Figure 2). Such workflows are a great help for the quantum chemist and can be executed in MoSGrid, for instance, with the quantum chemistry codes NWChem [41] or Gaussian09 [42]. With regard to the virtual screening of potential drugs, the submission of such a metaworkflow enables the efficient investigation of a larger number of molecules. After this basic characterization, further basic workflows can be added, for example, pKa calculation and charge decomposition analyses. The strength of the metaworkflow concept is that these further basic workflows can be added freely to the original metaworkflow, which even enhances the efficiency. This workflow can be executed several times in order to validate the molecules properties by simulations with various methodologies (different flavors of DFT).

## 3. Results 

The workflow illustrated in Figure 1 is the basis for our performance studies applying the DUD-E set for ABL1 (see above). As the preprocessing steps for the receptor such as the protonation state prediction and grid building are not influenced by the size and characteristics of the ligand library, our main focus was on the LigandFileSplitter and IMGDock applications. The first application is responsible for separating the ligand library into chunks of equal size. In this study we split the data into chunk sizes of 25, 50, 75, and 100 ligands, which corresponds to 448, 224, 150, and 112 individual IMGDock jobs. The pure wall time on the remote DCI was observed as well as data transfers between portal, DCI, and remote storage.

**Figure 3:**
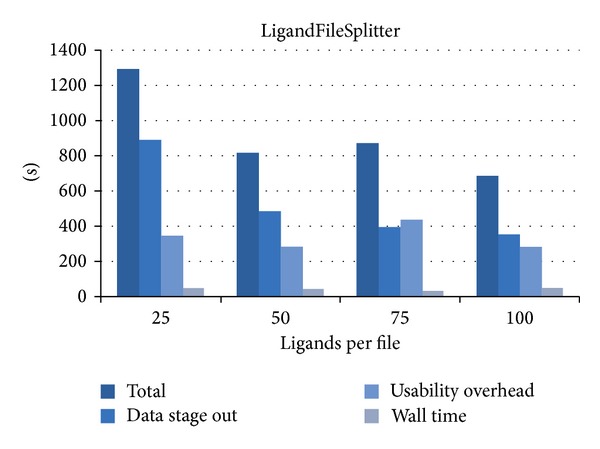
The performance of LigandFileSplitter for different sizes of data chunks.

Figure 3 illustrates the overall runtime of the LigandFileSplitter from the point of time the process is started via the science gateway until the point of time the results are transferred back to the science gateway. Additionally, we measured the times for staging out data from the computing resources to the science gateway and the wall time for the LigandFileSplitter on the computing resources. The time spent for the remaining tasks like staging the data and scheduling the job results via the subtraction of staging out the data and wall time from the total time is presented as usability overhead. The wall time of the process is almost the same for all runs independent of the number of files. LigandFileSplitter is a very short running task and has a wall time of about 50 seconds independent of the chunk size for a given number of ligands, for example, 11180 for ABL1. It consumes the smallest part of the overall runtime. The results show—as expected—that the staging of fewer but larger files consumes in general less time and the transfer of the files from the computing resources to the science gateway absorbs most of the overall time in general.

Since the times measured for usability features and data staging in the science gateway resulted in similar values for IMGDock and the response time in the science gateway scaled almost linearly over the whole workflow, we focused in the further performance studies on the wall time of IMGDock on the computing resources. The accumulated wall time for the docking lay between 301 and 444 computing hours and, thus, the added time via the science gateway infrastructure was insignificant because it ranges between 10 and 20 minutes. The surprising result was that IMGDock needed significantly less computing hours in total for more parallel jobs and smaller input files if all runtimes are added up including the scheduling time via the batch system. To exclude that this was a one-time event, we repeated all simulations a second time and a subset of simulations several times. While the second test run over all simulations confirmed the result, the test run on subsets has not shown this significant time difference but only a slight increase for the average time processing less jobs and larger files. However, the tendency of increased runtimes is clearly towards the smaller number of jobs. Since the docking of different ligands with different complexity also leads to different runtimes and, thus, to different results of the average time, our further analyses are based on the measures for the whole dataset. The values of Table 1 represent the wall time on the computing resource Atlas at Technische Universität Dresden, Germany, with its 92 nodes with each of the 64 cores (5,888 cores) over the complete dataset.

**Figure 4:**
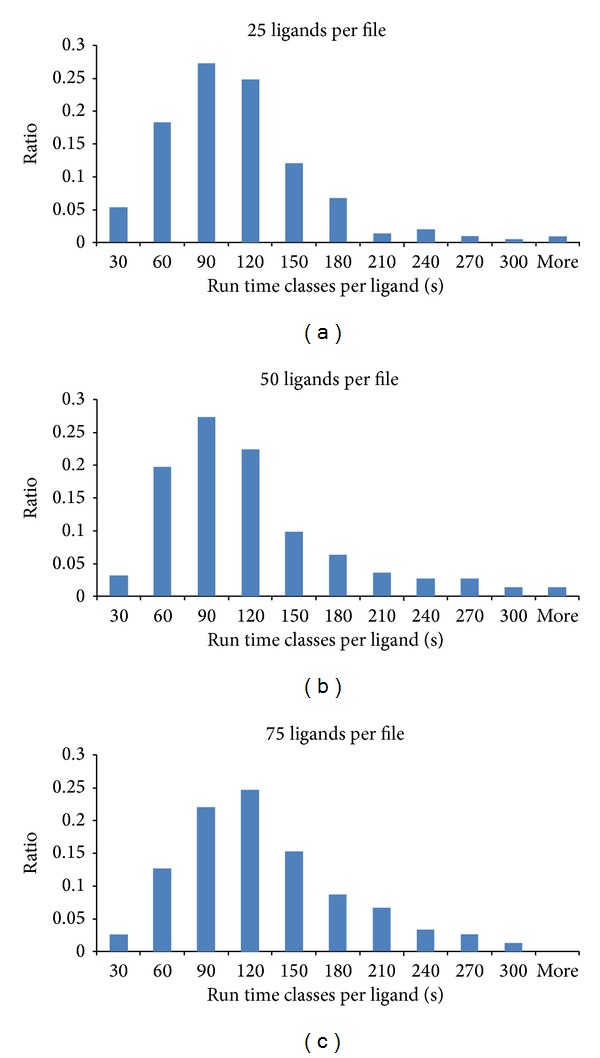
The distribution of the runtime classes of the docking runs with 25 ligands, 50 ligands, and 75 ligands is similar, which is also reflected in their runtimes for the whole process with the tendency to consume more time the larger the input files are.

**Figure 5:**
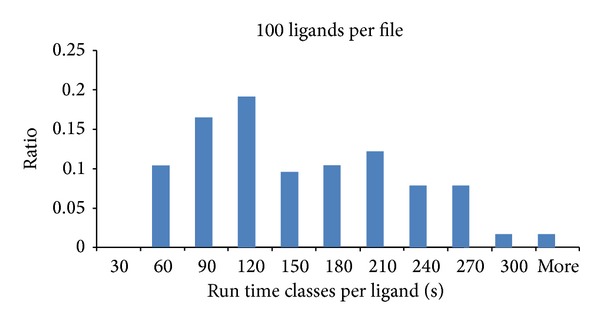
The distribution of the runtime classes of the simulations with 100 ligands differs significantly from the three other cases and reflects the increased runtime in this use case.

For further analyses we broke down the results into runtime classes per ligand. The runtime classes are divided into docking processes per ligand consuming 30 sec or less time up to 300 sec or more in steps of 30 sec (see Figures 4(a), 4(b), 4(c), and 5).

Since the pure docking process for the same ligands is expected to consume the same time, only the overhead of scheduling the job of reading the structure from files of different sizes and writing the results to files of different sizes can cause the difference.

To access the influence of the target protein and the characteristics of its binding site, five further datasets were created (see above), albeit with smaller ligand sets. The average wall time per ligand was as follows: acetylcholine esterase 55 sec; androgen receptor 20 sec; estrogen receptor beta 26 sec; HIV1 protease 105 sec; and thrombin 69 sec. Although the absolute values differ for each target, the characteristics of the runtime histograms resemble the observations reported for ABL1.

## 4. Discussion

In the age of big data, distributed infrastructures are increasingly complex and common at the same time. Via easy-to-use science gateways, users are enabled to make optimal use of these infrastructures to advance their respective state of the art. The infrastructures are hidden from the users enhancing their experience on the usability, while the added layers in the infrastructure by a science gateway affect also performance measures.

The individual runtimes are influenced by multiple factors. One factor is the extraordinary situation for a performance analysis that we on purpose have not used dedicated nodes for our studies. The goal was to simulate the real situation of users applying the MoSGrid science gateway for docking in their daily research. Whereas the overhead of the infrastructure adds a significant portion of time to jobs with small runtimes, this ratio is negligible for compute-intensive workflows. The most consuming task of the docking workflow is the docking method itself and IMGDock as serial application can be parallelized by splitting the data in meaningful chunks. Another factor, which influences the individual runtime, relates to the analyzed molecular structures and their features. The target protein and the characteristics of its binding site play a major role. While some pockets are rather small, preferring also small and rigid ligands, others are considerably larger. This leads to an increased number of degrees of freedom to be sampled, which consequently leads to longer runtimes.

Our studies have shown that the performance scales almost linearly for a growing number of jobs on the same datasets. The smallest unity benchmarked by us (25 ligands per file) is the one with the best performance even on wall time level and even though the larger number of jobs creates more overhead for managing the jobs. This surprising result on wall time level presumably results from a combination of factors: writing of larger files is also in ratio compared to smaller files much slower on the used file system (NFS in this case). Furthermore, all jobs are one-core jobs and the longer running ones are more often disturbed or interrupted than the short running jobs because of the parallel usage of the underlying compute nodes. Therefore, the optimal chunk size for splitting a docking library lies between 25 and 50 ligands with respect to the scientists' waiting time.

## 5. Conclusions 

Our performance studies show that the docking workflow for CADDSuite using the representative benchmark datasets for the protein kinase ABL1 with over ten thousand compounds scales almost linearly for up to 500 concurrent processes. The parallelization is achieved by splitting the data into optimal chunks for the docking task. The overhead for managing the parallel tasks and the overhead resulting from the science gateways infrastructure including the user interface layer and workflow system is negligible, while the science gateway especially adds usability aspects beneficial for the users. Although a particular docking tool was used, the results can be generalized for all applications available in the field. They will scale similarly even though the single runtimes of diverse docking applications will differ. In the future we intend to compare the runtimes of diverse docking tools like CADDSuite, FlexX, and AutoDock offered in the MoSGrid science gateway and the corresponding scientific results for various benchmark datasets.

## Figures and Tables

**Table 1:** Wall time distribution for IMGDock.

Jobs	448	224	150	112

Ligands per file	25	50	75	100

Wall time in hours	301	326	357	444

Average wall time in sec per job	2422	5239	8586	14275

Average wall time in sec per ligand	97	104	115	143
